# Phage display identifies two Caprine Arthritis Encephalitis Virus env epitopes

**DOI:** 10.1186/1297-9716-42-87

**Published:** 2011-07-22

**Authors:** Karlen Gazarian, Alvaro Aguilar Setién, Tatiana Gazarian, Sebastian Aguilar Pierle

**Affiliations:** 1 Departamento de Biología Molecular y Biotecnología, Instituto de Investigaciones Biomédicas, Universidad Nacional Autónoma de México (UNAM), México D.F., Ciudad Universitaria, 04510, México; 2 Unidad de Investigación Médica en Inmunología, Coordinación de Investigación IMSS Hospital de Pediatría 3er piso, Centro Medico Nacional Siglo XXI, Av. Cuauhtemoc 330, Doctores, México D. F., 06720 México; 3 Facultad de Medicina, Universidad Nacional Autónoma de México, (UNAM), México D.F., Ciudad Universitaria, 04510, México; 4 Department of Veterinary Microbiology and Pathology, Washington State University, Pullman, PO Box 647040 Pullman, WA, USA

## Abstract

Using phage display and IgG of a goat infected with Caprine Arthritis Encephalitis Virus (CAEV) we obtained families of 7 mer constrained peptides with consensus motifs LxSDPF/Y and SWN/KHWSY and mapped the epitopes mimicked by them at the Env 6-LISDPY-11 and 67-WNTYHW-72 sites of the mature gp135 amino acid sequence. The first epitope fell into the N-terminal immunogenic aa1-EDYTLISDPYGFS- aa14 site identified previously with a synthetic peptide approach; the second epitope has not been described previously. The first epitope is mostly conserved across CAEV isolates whereas the second newly described epitope is extremely conserved in Small Ruminant Lentiviruses env sequences. As being immunodominant, the epitopes are candidate targets for mimotope-mediated diagnosis and/or neutralization.

## Introduction, Methods and Results

Caprine arthritis-encephalitis virus, CAEV [1], and ovine maedi-visna virus, MVV [2] belong to the *Lentivirus *genus of the *Retroviridae *family. Since the first comparison in 1995 of *pol *gene sequences of French MVV isolates with CAEV [3] and subsequent extensive analyses (reviewed by [4]), studies have established that ovine MVV and caprine CAEV constitute a genetically heterogeneous group of pathogens that have evolved in small ruminants and are known as *small ruminant lentiviruses (SRLV*) [5-7]. Multiple observations support interspecies transmission of CAEV and MVV [5,8,9] in small domestic and wild ruminants, causing neurological, pulmonary, articular and mammary symptoms [1,4,10]. As transmission of CAEV repeatedly occurs in different regions [6,8,11,12], it affects herds worldwide and has a significant economic impact. The infection is clinically difficult to detect, hence sensitive diagnostic methods are of primary importance to prevent further distribution of the virus. Despite the availability of a large number of serological tests for CAEV [13], immunological detection is challenging due to the lack of low-cost and readily available recombinant antigens (see [14]). Testing sera with recombinant and synthetic peptides from different CAEV envelope (Env) regions permitted identification of several immunogenic regions [15-18]. Here we describe the first phage display mapping of epitopes of CAEV.

We used the VR905, CAEV 75-G63 strain, cloned lot 2D, 91-12, ATCC (American Type Culture Collection). Cell cultures of Mycoplasma-free goat synovial membrane (GSM) cells that had been propagated in Dulbecco-modified Eagle's medium (DMEM) containing 10% fetal bovine serum, penicillin 100 U/mL, streptomycin 100 mg/mL, and 2 µM L-glutamine were used. Goat synovial membrane cell monolayers were used for virus multiplication and were monitored twice a week for evidence of cytopathic effects (multinucleated giant cells, syncytia). The virus in cell supernatants was then titered through an endpoint dilution assay.

We infected a naïve 4-month old goat with the CAEV75-G63 strain. The goat was previously screened with a standard ELISA kit (CHEKIT CAEV/Maedi Visna Virus^®^, Behring, IL, USA) [19]). The goat was inoculated intravenously with 30 mL of GSM supernatant containing 1.5 × 105 TCID50 of CAEV. The titer was obtained indirectly by counting the number of syncytia. Goats infected with this inoculum consistently show signs of disease. Seroconversion of the infected goat can be detected by serological diagnosis (POURQUIER^® ^ELISA Maedi-Visna/CAEV; One IDEXX Drive Westbrook, Maine, New England, USA).

**Figure 1:**
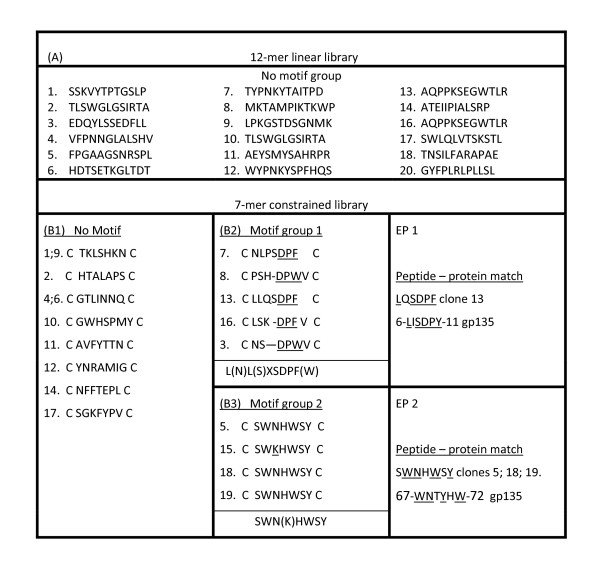
**Sequences of peptides retrieved by infected goat IgG from the 12 mer linear (A) and 7 mer constrained (B) random peptide libraries**. B1 - non-homologous peptides, B2 - homologous motif group 1 peptides, B3 - homologous motif group 2 peptides. Consensus sequences are shown in bottom of both groups. EP 1 and EP2 - CAEV Env sequences matching the motif sequences and considered the identified epitope sites (the SU mature protein amino acids numbering). Note: the clone 20 peptide shown in Figure 2 was not sequenced.

**Figure 2:**
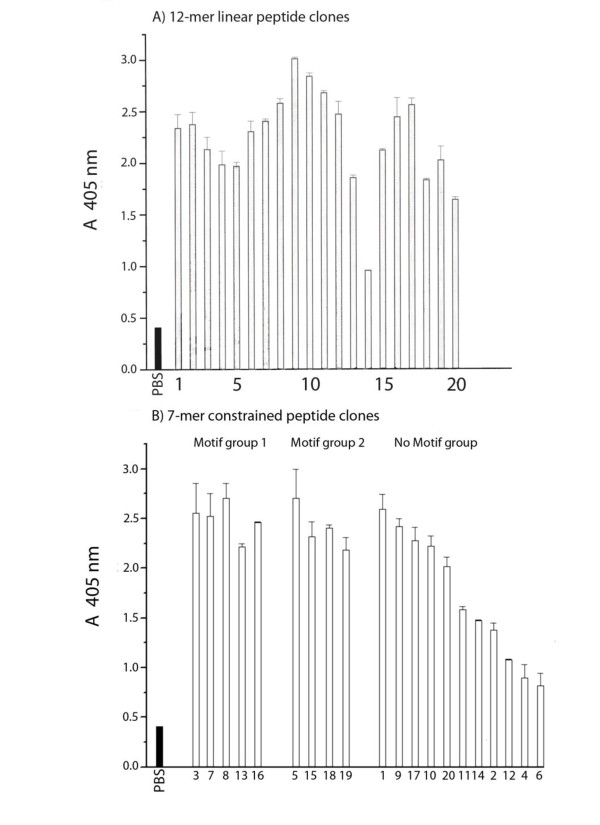
**Reactivity of the goat IgG with clones selected from the 12 mer linear (A) and the 7 mer constrained (B) libraries**. The shaded bars: PBS was added instead of the phage. Motif groups are shown.

IgG fraction purified from serum (see [20]) at 12-week post-infection (pi) was used to perform three-round panning of a 12 mer linear and a 7 mer constrained phage library (New England BioLabs Inc.,240 County Road, Ipswich, MA,USA) as previously described [20,21]. Twenty phage clones were isolated from each library; the sequences of DNA inserts in the phage pIII gene N-terminal region were determined using Sequenase kit and the amino acid sequences of peptides they encoded were deduced; reactivity of the IgG with phage clones was determined by standard phage ELISA [20,21]. Figures 1 and 2 depict the deduced sequences and reactivity levels shown by the selecting IgG with these clones in an ELISA test (clone no. 20 from the 7 mer library was tested for its reactivity but not sequenced). The shaded bars in Figure 2 show optical densities for wells containing PBS instead of IgG (blank). Clones with low signal (clone 14 from the 12 mer, and clones 12, 4, 6 from the 7 mer collections) serve as internal controls to indicate that the high optical density levels shown by the majority of the clones were due to reactivity with peptides and not phage antigens.

The deduced sequences of peptides from the 12-mer linear library did not show a detectable consensus motif (Figure 1a). Conversely, two motif groups designated as motif groups 1 and 2 (Figure 1 panels b2 and b3, respectively) comprising altogether 50% of the deduced sequences (the rest of sequences showed no similarity with other ones, Figure 1, b1) were derived from the 7 mer constrained library.

**Figure 3:**
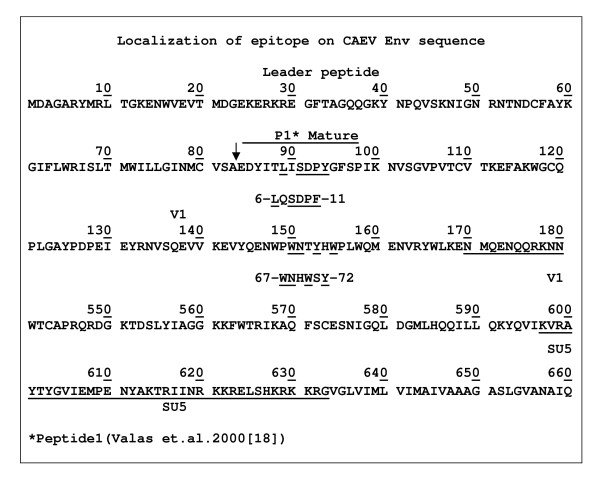
**Localization of epitopes on the CAEV Env N-terminal part of the mature protein**.

A homology search revealed two sites in the Env N-terminal region (Figure 3) matching the above mimotope motifs. One of these (EP1), with CAEV gp135 aa6-LISDPY-aa11 mature protein sequence, falls into the immunogenic amino acids 1 to 14 segment described by Valas et al. [18]. The second epitope (EP2), located in the mature protein aa67-WNTYHW-aa72 site and matching the mimotopes (SWNHWSY, Figure 1, clones 11,17,20) has not been described previously. Alignments and other bioinformatic analyses were performed using the CLC Protein Workbench version 3.0 (CLC bio), which aligns nucleotides and proteins using a progressive alignment algorithm [22], and performs secondary structure [23], antigenicity [24] and hydropathic [25] predictions. The first epitope is located at the very end of a beta strand and the second in a region that showed no particular structures but is adjacent to an alpha helix. The first epitope site was in a region whose antigenicity varied in different isolates from -0.04 to -0.02. For the second epitope site the values ranged from -0.03 to -0.04. The predicted hydrophobicity for the first epitope site was significantly higher (from 1.5 to 1.9 in the isolates) than for the second epitope (-2.0 to -2.1). Sixteen different origin SRLV Env aligned sequences (Additional file 1) show that the second newly described epitope is extremely conserved across SRLV sequences. In contrast, the first previously described epitope is mostly conserved across CAEV isolates.

## Discussion

Previous work by Bertoni's [16,17] and Valas' [18] groups with recombinant and synthetic peptides describe several multi-epitope immunogenic regions throughout the length of Env subunit SU. In this study, we took advantage of the following characteristics of high throughput phage display epitope mapping [26]: (1) apart from identification of epitopes, it highlights their crucial amino acids; (2) it provides families of antigenic mimics, mimotopes, that may be used as novel diagnostic and antiviral antigens, (3) the epitopes identified are very likely to be immunodominant (gauged by the appearance of its mimics in 50% of the 19 sequenced clones randomly picked from the screened 7 mer library of 10^8 ^phages). Two libraries with different peptide lengths and conformation were used. As shown by our previous screenings, one of the libraries can mimic an epitope more successfully than the other depending on its structure [20,21]. Here the 7 mer cyclic peptides mimicked the epitopes more adequately. To obtain serum-containing antibodies elicited by immunodominant epitopes, we infected a goat with CAEV. Although goats infected with CAEV75-G63 mount an early (3 to 4 weeks) response to Gag and Env, seroconversion to immunodominant epitopes such as TM3 [15], a homologue of the HIV-1 principal immunodominant epitope [20,26] was shown to delay significantly, appearing between week 12 and 28 pi. Considering this, we took serum at 12 weeks pi and obtained antibodies that successfully selected groups of five and four mimics of epitopes 1 and 2, respectively. Our data show that seroconversion took place when using immunodominant epitope Ep2 at 12-weeks pi.

The coincidence of one of our mapped epitopes, aa6-LISDPY-aa11, with the previously identified immunogenic aa1- aa14 site [18] validates our selection results. The second epitope, aa-67 PWNTYHW-aa72, has not been described previously. This epitope was not precisely mapped in Bertoni's experiment due to the large size of peptides they used as antigens [16].

In the experiments by Valas et al. [18], peptide No. 10 with aa64-aa77 sequence covering our epitope aa67-aa72 sequence was recognized by 4% of the tested sera but in their study the criterion for the immunodominance was the number of positive immune sera to an individual peptide and not the dominance of the antibody in the serum which is the criterion in phage display mapping. Hence, the authors focused on the peptides recognized by more than 10% of sera. Both of these epitopes fell within a relatively short (72 aa) N-terminal section of the mature protein corresponding to SU1 and SU2 [16]. The fact that phage display did not detect the earlier reported [16,17] C-terminal SU5 immunogenic domain (underlined in Figure 3) confirms the relevance of the conformational structure of epitopes in this region because the method experiences difficulties with this type of epitope [21] (reviewed by [27]).

In conclusion, this first phage display study describes two discrete epitopes on CAEV gp135 N-terminal segment preceding the first variable V1 domain of the protein. One of them coincides with the previously reported N-terminal immunogenic site; the other was discovered for the first time. Apart from precise mapping of these epitope cores, the study highlights their critical (contact) amino acids and some secondary structure features. In forthcoming experiments the epitopes will be subjected to functional analysis to determine the potentials of their mimotopes for diagnosis of the infection and for virus neutralization (as done for HIV-1 [26,28]).

## Competing interests

The authors declare that they have no competing interests.

## Authors' contributions

KG conceived the study, and participated in its design, coordination, contributed to the analysis of the results and preparation of initial and revised manuscript versions. AGS participated in designing of the experimental strategy, carried out the goat immunization, obtaining and characterization of sera; TG carried out the biopanning experiments, immunization of mice with mimotopes and immuno-testing the anti-mimotope sera; SAP - carried out the computer analysis of selected peptides and Env sequences, contributed to the preparation of the manuscript. All authors read and approved the final manuscript.

## Supplementary Material

Additional file 1 **Alignment of 16 env sequences showing the Ep1 (red) and Ep2 (yellow) sequence conservation**. Click here for file
