# Supplementary material for: Phage display identifies two Caprine Arthritis Encephalitis Virus env epitopes
Source: Vet Res. 2011 Jul 22;42(1):87. doi: 10.1186/1297-9716-42-87 (PMC3162499; doi:10.1186/1297-9716-42-87)
Supplement: Additional file 1 — Alignment of 16 env sequences showing the Ep1 (red) and Ep2 (yellow) sequence conservation. [file 1297-9716-42-87-S1.DOC]

1 50

Cork (1) --------MDAGARYMRLTGKENWVEVTMDGEKERKR--EGFTAGQQGKY

Clements (1) --------MDAGARYMRLTGKENWVEVTMDGEKERKR--EGFTAGQQGKY

CAEV63 (1) --------MDAGASYMRLTGEENWVEVTMDEEKERKG--K---DVQQGKY

Gansu (1) --------MDAGASYMRLTGEENWAEVTMDEEKERKG--K---DVQQGKY

Shanxi (1) --------MDAGARYMRLTGEENWVEVTMDEEKERKG--K---DVQQGKY

Chi3056 (1) --------MDAGAKHMRFTEEENWSEVTMEEVEERKG--EGFTKRQPGRY

SRLV B 1 MX (1) --------MDAGAKHIRFTGEETWCEVTMGEEGKKKQ--EGCNKEQQGKY

Ov496 (1) --------MDMGAKHMQRTGEGNWVEVKMEEQEREEE--PLLHTRQQGKY

Roccaverano (1) ---------------MDRRLRKQWMEGVQ-------------------AY

S7049AW (1) ------------------------MERSQEK-------------E---KE

S93 (1) MAS-KNRPSRVTWKGMEPPLRETWNEVLNELVQKHQE--GEQETLIAGKQ

EV1 (1) MASTKSKPSRATWADMEPPQEEKWGQVVQELVTRQQN--EERQGLVTGKR

SA-OMVV (1) MASSKNMPSRITQKSMEPPLRETWQQVVQEMVMRKQRDEEEKQNLVTGKE

1514 (1) MTSKESKPSRTTWRGMEPPLRETWNQVLQELVKRQQQEEEEQQGLVSGKK

S747ST (1) MASTRSKPSRTNWSAMEPPLKETWNHVVQELVKRQQNQEEEEIGLVTGKK

S8012VE (1) MASTRSKPSRGTWPTMEPPLKETWNQVVQEMVKRQQSQNEEEIGLVTGKK

Consensus (1) MDAGAKYM T KENW EVTME KRR E LQ GKY

51 100

Cork (41) NPQVSKNIGNRNTNDCF--------------AYKGIFLWRISLTMWILLG

Clements (41) QPQVSKQIGNRNTNPCF--------------AYKGIFLWRISLTMWILLG

CAEV63 (38) RPQVSKPIINRDTNTSF--------------AYKGIFLWGIQITMWILLW

Gansu (38) RPQVSKPIINRDTNTSF--------------AYKGIFLWGIQITMWILLW

Shanxi (38) RPQVSKPIINRDTNTSF--------------AYKGIFLWGIQITMWILLW

Chi3056 (41) TPQVSTQIEERDTNTRF--------------AYTSMFLWGIQLTVWIVLL

SRLV B 1 MX (41) RLQVSTNIC--------------------------LIYT------CILLW

Ov496 (41) KPQVSEQILNGDTNTCL--------------TYAWIFLWGIQLTLWILLL

Roccaverano (17) KGKVNIQVS----------------------------LWVKVIIGASMVG

S7049AW (11) RMIQGKCQYQVS-----------------------------RISLVFLIV

S93 (48) KSWVSIDLLGTEQREDKRKINVWEPCERWFAQVIWEVLWVIQIMLWGCLL

EV1 (49) KPWVSIEILGTNHDQEKEKVNVWEPCEKWFGQLVWVTLWMLQLVLWGCLI

SA-OMVV (51) KSWVSIDLLGTKQEGKRQKVNIWGPWEKWGIKIVWVMLWVIQLMLWGCLI

1514 (51) KSWVSIDLLGTEGKDIK-KVNIWEPCEKWFAQVVWGVLWVLQIVLWGCLM

S747ST (51) RSWVSIDIMGTDREEVEQKVNIWEPCEKWGAKVIWAILWVIQIILWGCLI

S8012VE (51) RSWVSIDLMGTDREEGERKVNIWEPCEKWGAKVIWGILWVIQIMLWGCLI

Consensus (51) RPQVS IL D I LW IQITLWILLI

101 **>1< Vs1/ep1** 150

Cork (77) IN------MCVSAEDYITLISDPYGFSPIKNVSGVPVTCVTKEFAKWGCQ

Clements (77) IN------MCVSAEDYITLISDPYGFSPIKNVSGVPVTCVTKEFAKWGCQ

CAEV63 (74) TN------MCVRAEDYITLISDPYGFSPIKNVSGVPVTCVTKEFARWGCQ

Gansu (74) TN------MCVRAEDYITLISDPYGFSPIKNVSGVPVTCVTKEFARWGCQ

Shanxi (74) TN------MCVRAEDYITLISDPYGFSPIKNVSGVPVTCVTKEFARWGCQ

Chi3056 (77) SQGPH---MYVSAEEYITLISDPYGFSPVRNVSGVPVTCVTKEFAKWGCQ

SRLV B 1 MX (59) IS------MCASAEDYITLISDPYGFSPVRNVSGVPVTCMTKEFAKWGCQ

Ov496 (77) LQWPS---MGCKAEDYITLISDPYGFQPVRNVSGVPVTCVTKKFAKWGCQ

Roccaverano (39) SG--------SVGEEYITMVSDPYGFKPIWDVTNVPVTCVTKNFTQWGCQ

S7049AW (32) IEA-------VQGVDYITLISDPYGFSPVKNVSGVPVTCITKEFSKWGCQ

S93 (98) WEVNKTSG--CKAEEVIALIDDPGGFQKVTQVETVPVTCTTKNFTQWGCQ

EV1 (99) WEMQKESKGQCQAEEVIALIDDPGGFQRVRQVETVPVTCVTRNFTQWGCQ

SA-OMVV (101) WEMGKKH--SCNAEEVIALVDDPGGFQKVKYVESVPVTCMTKNFTQWGCQ

1514 (100) WEMRKGN--QCQAEEVIALVSDPGGFQRVQHVETVPVTCVTKNFTQWGCQ

S747ST (101) WEMQKDNR-QCKAEEVIALVDDPGGFQRVKQIETVPVTCVTKNFTQWGCQ

S8012VE (101) WEMQKTNS-QCKAEEVIALVDDPGGFQRVKQIATVPVTCVTKNFTQWGCQ

Consensus (101) M AEDYITLISDPYGFSPVKNVSGVPVTCVTKEFAKWGCQ

Mimotope **LQSDPF**

151 **ep2** 200

Cork (121) PLGAYPDPEIEYRNVSQEVVKEVYQENWPWNTYHWPLWQMENVRYWLKEN

Clements (121) PLGAYPDPEIEYRNVSQEVVKEVYQENWPWNTYHWPLWQMENVRYWLKEN

CAEV63 (118) PLGAYPDPEIEYRNVSQEIVKEVYQENWPWNTYHWPLWQMENVRYWLKEN

Gansu (118) PLGAYPDPEIEYRNVSQEIVKEVYQENWPWNTYHWPLWQMENVRYWLKEN

Shanxi (118) PLGAYPDPEIEYRNVSQEIVKEVYQENWPWNTYHWPLWQMENVRYWLKEN

Chi3056 (124) PLGAYPDPEIEYRNVSQEIVKEVYQEDWPWNTYLWPLWQMENVRQWLKEN

SRLV B 1 MX (103) PLGAYPDPEIEYRNVSQEILKEVYRNNWPWNTYHWPLWQMENVRQWLKET

Ov496 (124) PIGAYPNPEIEYRNISSDILKEVYQEDWPWNTYHWPLWQMENVKQWLKEN

Roccaverano (81) PEGAYPDPQKEYRNMSTDILEEVYNRTWPWRTYHWPLWQMDNMRRWAEQN

S7049AW (75) PIGAYPDPDLEYRNISNEILEEVYQKDWPWNTYHWPLWQMDNVVQWARQN

S93 (146) PEGAYPNPEVEYRNISQDILKEVYKDEWPWNTYHWPLWQMENMRQWMKEN

EV1 (149) PEGAYPDPEIEYRNISKEILEQVYGRDWPWNTYHWPLWQLENMKSWMKEN

SA-OMVV (149) PEGAYPDPDLEYRNISQDILEQVYKQEWPWNTYHWPLWQMENMRQWMKEN

1514 (148) PEGAYPDPELEYRNISREILEEVYKQDWPWNTYHWPLWQMENMRQWMKEN

S747ST (150) PEGTYPNPEEEYRNISRDILEEVYKRDWPWNTYHWSLWQMENMRQWMKEN

S8012VE (150) PEGAYPDPEVEYRNISQNILEEVYKQDWPWNTYHWPLWQMENMRQWMKEN

Consensus (151) PLGAYPDPEIEYRNISQEILKEVYQEDWPWNTYHWPLWQMENVRQWLKEN

Mimotope **WNHWSY**

201 **V1** 250

Cork (171) MQENQQRKNNTKEGIEELLAGTIRGRFCVPYPFALLKCTKWCWYTAAINN

Clements (171) MQENQQRKNNTKEGIEELLAGTIRGRFCVPYPFALLKCTKWCWYTAAINN

CAEV63 (168) IAENKKRKNSTKKGIEELLAGTIRGRFCVPYPFALLKCTKWCWYPAEIDQ

Gansu (168) IAENKKRKNSTKEGIEELLAGTIRGRFCVPYPFALLKCTKWCWYPAEIDQ

Shanxi (168) IAENKKRKNSTKEGIEELLAGTIRGRFCVPYPFALLKCTKWCWYPAEIDQ

Chi3056 (174) EKEYRQRKNNTKEGMDDLLAGKIRGRFCVPYPFALLKCTEWCWYPAEIDK

SRLV B 1 MX (153) EKE--RKENSTKEGIDKLLSGQIRGRFCVPYPFALLKCTEWCWYPAVIDK

Ov496 (174) EKDYKRKLNTTKEDLDELLAGKIRGRFCVPYPFALLKCTEWCWYPAAIDD

Roccaverano (131) LQD-----NKTRPQIEQLLAGQIRGKFCVPYPYAMLRCEQWCWY----KA

S7049AW (125) LQGDK----KEKRDLADLLAGKIRGRFCVPYPFALLECMEWCWWVKGTDA

S93 (196) EKEYKGRTNKTKEDIDALLAGKIRGRFCVPYPYALLRCTKWCWYPANIDE

EV1 (199) EKENKGRTNKTKEDIDDLLAGRIRGRFCVPYPYALLKCEEWCWYPTDINE

SA-OMVV (199) EKEYTSRNNKTKEDIDALLAGKIRGRFCVPYPFALLKCEEWCWYPANINQ

1514 (198) EKEYKERTNKTKEDIDDLVAGRIRGRFCVPYPYALLRCEEWCWYPESINQ

S747ST (200) EGEYKGRTNKTKEDIDDLLAGKIRGRFCVPYPFALLKCEEWCWYPSRINQ

S8012VE (200) EKEYTGRTNKTKEDIDDLLAGKIRGRFCVPYPFALLKCEEWCWYPSQINE

Consensus (201) EKE K R NKTKEDIDDLLAGKIRGRFCVPYPFALLKCTEWCWYPA IDQ

251 300

Cork (221) ESGKAGKIKINCTEARAVSCTEDMPLASIQRAYWDEKDRESMAFMNIKAC

Clements (221) ESGKAGKIKINCTEARAVSCTEDMPLASIQRAYWDEKDRESMAFMNIKAC

CAEV63 (218) ETGRARKIKINCTEARAVSCTEEMPLASIHRAYWDEKDRESMAFMNIRAC

Gansu (218) ETGRARKIKINCTEARAVSCTEEMPLASIHRAYWDEKDRESMAFMNIRAC

Shanxi (218) ETGRARKIKINCTEARAVSCTEEMPLASIHRAYWDEKDRESMAFMNIRAC

Chi3056 (224) ESGRAEKIKINCTEVRAVSCTEDMPLASIRRAYWDEKDRESMAFMNIQAC

SRLV B 1 MX (201) ESGQARKIKINCTKARAVSCTEEMPLASVHRAYWERRDKESMAFMNIKAC

Ov496 (224) ESGRAKNIKINCTNARAVSCTEEMPLASIHRAYWGKRERESMQFMNIRAC

Roccaverano (172) EE--E--IRIDCTRAVAVSCTNELPLAGLKRVYWEEKDRQDMEFMNIKAC

S7049AW (171) WGYGEADIRINCSQARAVSCTHEMPLASVQRVYWSREDKGAMERMIIKPC

S93 (246) ESGRARKIKINCSEARAVSCTEVMPLASIHRAYWDKPDKKSMEFMNIEAC

EV1 (249) ETGHAQKIKINCTKAKAVSCTEKMPLAGVQRVYWEKEDEESMKFMNIEAC

SA-OMVV (249) ETGHAQQIKINCTKAKAVSCTEQMPLAAVQRVYWEKEDEEGMKFMNIQAC

1514 (248) ETGHAEKIKINCTKAKAVSCTEKMPLAAVQRVYWEKEDEESMKFLNIKAC

S747ST (250) ETGHAEQIKINCTKAKAVSCTEKMPLASVQRVYWEKEDEESMKFLNIQVC

S8012VE (250) ETGHAEKIKINCTKAKAVSCTEKMPLAAVQRVYWEKEDEESMRFLNIEAC

Consensus (251) ETGRA KIKINCT ARAVSCTEEMPLASIQRAYWDKKDRESM FMNIKAC

**Accession numbers for sequences used in the alignment are:**

**1. Cork** P31626.1 **9**. **Roccaverano** ACA81613.1

**2. Clements** AAA91829.1 **10**. **S7049AW** ABI29235.1

**3. CAEV63** AAB88709.2 **11**. **S93** AAK82340.1

**4. Gansu** AAX81904.1 **12**. **EV1** ABO32371

**5. Shanxi** ACY78388 **13**. **SA-OMVV** AAA66817.1

**6. Chi3056** ABA39071 **14.** **1514** P23423

**7. SRLV B1 Mexico** HM210570.1 **15**. **S747ST** ABI29241

**8. Ov496** ACN82425.1 **16.** **S8012VE** ABI29242

………………………………………………………………………………………………………

**Explanations**:

**>1<** Leader peptide – mature protein junction.

**Vs1/ep1** Epitope 1 (ep1) core sequence (red) mapped into the N-terminal 14 amino acid

immunogenic site [18].

**ep2** Epitope 2 core sequence (yellow).

**V1** Env variable 1 region.
